# Quantitative Analysis of Muscarinic Antagonist Atropine and Tiotropium in Microvolume Plasma Using Liquid Chromatography–Mass Spectrometry: Application for Pharmacokinetic Studies

**DOI:** 10.1155/jamc/9923229

**Published:** 2025-04-08

**Authors:** Marilène Trancart, Mylène Penot, Gwladys Meesemaecker, Romain Boffy, Anne-Sophie Hanak, André-Guilhem Calas, Nicolas Taudon

**Affiliations:** ^1^Toxicology and Chemical Risks Department, French Armed Forces Biomedical Research Institute, Brétigny-sur-Orge, France; ^2^Analytical Development and Bioanalysis Unit, Platforms and Technological Research Department, French Armed Forces Biomedical Research Institute, Brétigny-sur-Orge, France

**Keywords:** atropine, LC-MS/MS, medical countermeasure, organophosphorus compounds, pharmacokinetics, plasma, tiotropium

## Abstract

Despite the availability of current resources, the development of medical countermeasures remains crucial in combatting the threat posed by chemical warfare agents, such as organophosphorus compounds (OPs), which are toxic nerve agents requiring rapid medical intervention. Within the available therapeutic arsenal, muscarinic antagonists such as atropine are administered to mitigate the effects of excessive cholinergic system stimulation, which leads to respiratory tract obstruction due to hypersecretions and bronchoconstriction. Tiotropium, an FDA-approved bronchodilator, acts as a muscarinic receptor antagonist and could, therefore, serve as a potential alternative. To assess its potential efficacy in attenuating OP-induced respiratory effects in a murine intoxication model, it was necessary to first characterize its pharmacokinetic properties. A liquid chromatography-mass spectrometry method was developed and validated following ICH M10 guidelines for the quantification of atropine and tiotropium in 10 μL of plasma. The sample pretreatment procedure involved solid-phase extraction. Chromatographic separation was achieved using a fully porous sub 2 μm C18 column. The analysis was completed in just 4 min, with analytes identified and quantified using two selected reaction monitoring transitions. The mean extraction recoveries exceeded 90% for both drugs, and no matrix effect was observed. The lower limits of quantification were 0.5 ng/mL for tiotropium and 1.0 ng/mL for atropine, with an upper limit of 1000 ng/mL. The signal-to-concentration ratio demonstrated recoveries of back-calculated concentrations ranging from 94% to 108% (relative standard deviation (RSD) < 9.0%). Within-run and between-run precisions were both below 8% with accuracies ranging from 87% to 110%. This highly specific and sensitive method has proven useful for analyzing samples from pharmacokinetic studies conducted in mice. Following intraperitoneal administration, the AUC for tiotropium was approximately twice that of atropine, while its Tmax was half as long (4.9 vs. 8.2 min). The terminal half-lives were approximately 7.5 min for tiotropium and 9.8 min for atropine.

## 1. Introduction

Organophosphorus compounds (OPs), primarily used as pesticides [1], have proven to be extremely toxic, leading to their use as chemical warfare agents. Also known as nerve agents, OPs have been employed in armed conflicts, political assassinations, and terrorist attacks. Their development, production, stockpiling, and use have been prohibited since 1997 under the Chemical Weapons Convention. However, the current unstable geopolitical climate, characterized by the threat of high-intensity conflict and terrorist acts, underscores a need for effective countermeasures against this chemical threat. Indeed, since the 2010s, there has been a resurgence in the use of these agents [2–7].

These compounds are considered neurotoxic as they disrupt nerve impulse transmission by irreversibly inhibiting cholinesterase enzymes, specifically acetylcholinesterase (AChE, EC 3.1.1.7) and butyrylcholinesterase (BChE, EC 3.1.1.8). These enzymes are responsible for the degradation of the neurotransmitter acetylcholine. When acetylcholine is no longer hydrolyzed at the synaptic cleft, it results in the overstimulation of muscarinic and nicotinic receptors, followed by the desensitization of nicotinic receptors [8]. Ultimately, exposure to OPs leads to a severe cholinergic crisis. In the absence of prompt and adequate medical care, death from such exposure is primarily due to respiratory failure, culminating in hypoxemia and consequent cardiopulmonary arrest [9, 10].

The current medical management primarily relies on the administration of atropine, a muscarinic receptor antagonist, to reduce receptor overstimulation. [11]. However, while treatments for OP poisoning exist, they do not provide complete protection against the entire spectrum of neurotoxic threats. Therefore, improving this therapeutic framework remains a topic of current interest and active research [12, 13].

Safeguarding respiratory function is a critical aspect of addressing this challenge. Given its prevalence in the respiratory system and its role in airway smooth muscle contractility [14], antagonizing the M3 muscarinic receptor subtype is a relevant therapeutic approach for managing respiratory toxicity [15]. Tiotropium, an FDA-approved bronchodilator used in the treatment of chronic obstructive pulmonary disease (COPD), acts as a muscarinic receptor antagonist with a specific affinity for the M3 subtype [16]. To assess its potential efficacy in attenuating OPs-induced respiratory effects, it is crucial to determine the pharmacokinetic profile of tiotropium. Blood sampling at different time points is required.

**Figure 1:**
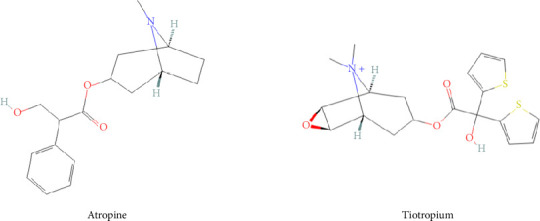
Chemical structures of atropine and tiotropium.

The quantification of alkaloids, particularly atropine, by liquid chromatography-mass spectrometry (LC-MS/MS) has been extensively studied. However, most validated analytical methods require sample volumes (100–300 μL [17–21]) that are not compatible with our murine model, which is nonetheless essential for screening candidate compounds, particularly against OP exposure. To minimize the number of experimental animals, blood collection is performed via the saphenous vein, allowing repeated measurements in the same individual. However, this approach does not yield the blood volumes typically required by standard quantification protocols described in the literature. Obtaining such volumes in our model would necessitate terminal sampling after euthanasia, leading to an excessive number of animals to cover all time points, raising significant ethical concerns. To address this limitation, we have developed and validated an LC-MS/MS-based quantification method optimized for very small plasma volumes (10 μL), thereby reducing the number of animals while ensuring reliable and reproducible quantification. This method allows for the establishment of the blood pharmacokinetic properties of tiotropium and atropine (Figure 1) in mouse samples.

## 2. Materials and Methods

### 2.1. Chemicals and Reagents

Atropine sulfate monohydrate (MW = 578 g mol^−1^, purity > 99%) was supplied by the Central Army Pharmacy (Orléans, France). Tiotropium bromide monohydrate (MW = 490.43 g mol^−1^, purity > 98%) and ammonium formate 10 M were purchased from Sigma-Aldrich (Saint-Quentin-Fallavier, France). Atropine-d_5_ (MW = 294.4 g mol^−1^, purity > 99%) and tiotropium-d_3_ bromide (MW = 475.4 g mol^−1^, purity > 99%) were purchased from Bertin Bioreagent (Montigny-le-Bretonneux, France). Acetonitrile, methanol, and formic acid (LC-MS grade) were obtained from Biosolve Chimie (Dieuze, France). Ultrapure water (UPW) was obtained from a Milli-Q IQ 7003 water purification system (Millipore, Bedford, USA).

### 2.2. Chromatographic and Spectrometric Conditions

The liquid chromatography (LC) system consisted of an Exion LC coupled to a 6500 + QTRAP mass spectrometer (Sciex, Villebon-sur-Yvette, France) equipped with an electrospray source operating in positive ionization mode (ESI+). The mass spectrometer (MS) parameters were as follows: ion source temperature, 500°C; ion-spray voltage, 3000 V; nebulizer gas flow (ion source gas 1, GS1), 50 psi; heater gas flow (ion source gas 2, GS2), 50 psi; and curtain gas (CUR), 25 psi. The dwell time for each transition was fixed at 40 ms. Fragments were formed under medium collision-activated dissociation gas (6V). Data acquisition was performed in multiple reaction monitoring (MRM) mode. The specific parameters for atropine, tiotropium, and their internal standard (IS) are detailed in Table 1.

The separation of the analytes was performed using an Acquity BEH C18 column (50 × 2.1 mm, 1.7 μm) protected by a BEH C18 Vanguard precolumn, both obtained from Waters (Saint-Quentin-en-Yvelines, France). Their temperature was maintained at 40°C. The injection volume was 1 μL, with a flow rate of 0.5 mL/min. The mobile phase consisted of phase A (5 mM ammonium formate buffer in UPW adjusted to pH 3.5 with formic acid) and phase *B* (formic acid 0.1% *v*/*v* in acetonitrile). The gradient elution began with the mixture of 85% phase *A* and 15% phase *B*. From 0 to 2.5 min, phase *A* was linearly decreased to 55%. From 2.5 to 3.5 min, phase *B* was increased to 95% and maintained for 1 min. The initial conditions were restored within 0.5 min. The total run time was 4 min, followed by a 2 min postrun re-equilibration phase.

The LC-MS system was controlled using the Analyst software (version 1.7.3), and data processing was performed using the SciexOS software (version 2.0.0).

### 2.3. Preparation of Calibrators and Quality Control Samples

Atropine and tiotropium were dissolved in N, N-dimethylformamide (DMF) to prepare 1 mg/mL of stock solutions, which were aliquoted and stored at −80°C. Two independent solutions were prepared for calibrators and quality control (QC) samples for each compound. For each drug, eight standard working solutions were prepared extemporaneously by appropriately diluting the stock solutions in a water-methanol mixture (50:50, *v*/*v*), with concentrations ranging from 0.010 to 20 μg/mL for tiotropium and 0.020–20 μg/mL for atropine.

The IS, atropine-d_5_, and tiotropium-d_3_ were initially dissolved in DMF to obtain 1 mg/mL of stock solutions. Prior to use, these stock solutions were thawed, diluted in a 25-mM formate buffer to a final concentration of 36 pg/mL, and subsequently used for sample dilution.

To prepare calibrators and QC samples, 5 μL of the working solution was added to 95 μL of a drug-free plasma. The calibration range consisted of eight points, spanning concentrations from 0.5 to 1000 ng/mL. QC samples were prepared at four concentration levels: 0.5 and 1 ng/mL for tiotropium and atropine (lower limit of quantification QC samples), respectively; 1.5 ng/mL (low QC samples); 50 ng/mL (medium QC samples); and 750 ng/mL (high QC samples).

### 2.4. Sample Preparation Procedure

The sample purification process involved a weak ion exchange solid phase extraction on Oasis WCX cartridges 1 cc/30 mg purchased from Waters. Extraction was performed in the positive pressure mode (Pressure + 48, Biotage, Uppsala, Sweden).

A volume of 290 μL of IS solution was added to 10 μL of the study, calibration, and QC samples. The mixture was deposited on a cartridge previously conditioned with 1 mL of acetonitrile followed by 1 mL of purified water. The washing step consisted of adding 1 mL of 25 mM ammonium formate buffer followed by 1 mL of acetonitrile. Analytes were eluted in a 5-mL polypropylene tube with 1 mL of acetonitrile containing 5% of formic acid. The extract was evaporated to dryness under vacuum at 125 mbar for 40 min at 40°C using a Genevac EZ-2 4.0 system (Biopharma Technologies France, Diémoz, France). The dry residue was dissolved in 75 μL of a water/acetonitrile mixture (50:50, *v*/*v*) containing 0.1% of formic acid, by vortexing for 5 min at 800 rpm before being transferred to a vial for analysis.

### 2.5. Validation Procedure in Human Plasma

To limit the use of the animal matrix, a full validation was first performed in human plasma before transferring the method to murine plasma. In compliance with French regulations, plasma was obtained under an agreement with Etablissement Français du Sang (Rungis, France) from healthy volunteers not undergoing drug therapy. Validation was performed according to the ICH M10 Bioanalytical Method Validation Guideline [22].

Selectivity and specificity: The selectivity and specificity of the analytical method were evaluated using six individual plasma batches, spiked with and without compounds at their lower limit of quantification (LLOQ) and their corresponding IS. The retention times of endogenous compounds in the matrices were compared with those of the target compounds to assess potential interferences. The absence of interfering components was confirmed when the signal in the blank matrix remained below 20% for the drugs and below 5% for their respective IS.

Drug/response relationship: The analytes' peak areas were normalized to those of the IS and plotted against their respective concentrations. The relationship between the nominal analyte concentration and the area ratios was assessed across three independent runs, performed on different days using three distinct matrix batches. Linear and quadratic regression models, unweighted or weighted by analyte concentration, were tested. Regression curves were not forced through zero. Discrepancies between nominal and back-calculated concentration were investigated by analyzing the distribution of residuals (differences between nominal and back-calculated concentrations), and mean residual (mean predictor errors) values were computed and compared to zero (Student's *t*-test).

Within-run precision and accuracy: Precision and accuracy were determined for both within-run and between-run assays using QC samples at all concentration levels (LLOQ, low, medium, and high). For the within-run assay, five independent replicates were analyzed. For the between-run assay, two replicates were analyzed daily over six consecutive days. Precision was expressed as the relative standard deviation (RSD). Accuracy was expressed as a percentage of the nominal concentration.

Matrix effect and extraction recovery: The matrix effect and recovery were evaluated using low and high QC samples across six different matrix batches (*n* = 3 per concentration and matrix batch, i.e. *n* = 18 per concentration level). For the matrix effect, the analyte response was assessed by contrasting the peak area ratio of reconstituted extracts, that is blank plasma spiked with reference solution after the extraction step, with those from the reference solutions, aiming for equal ratios (100 ± 15%) for the RSD below 15%. The areas under the peaks of extracted QC samples were compared with those of the reconstituted extracts to characterize the absolute extraction recovery. A minimum extraction percentage was not defined as an acceptance criterion. However, an RSD below 15% was expected.

### 2.6. Matrix Effect and Extraction Recovery

Carry-over: Carry-over was evaluated at analyte and IS retention times by analyzing blank samples following the upper limit of quantification (ULOQ) from each calibration curve (*n* = 6). The signal intensity had to be below 20% for the drugs and below 5% for their respective IS.

Analyte stability: Stability evaluation of stock and working solutions of the analyte and IS were carried out under conservation (−80°C, 4°C) and working (on the bench) conditions. In the same way, the stability evaluation of samples was carried out under conservation (−80°C, −30°C) and working (on the bench, 4°C, freeze and thaw) conditions. Finally, the stability of the processed samples at room temperature (on the bench) and 6°C (on an autosampler) was studied. All stability tests in the matrix were performed at two concentration levels of QC samples: low and high, with three replicates for each; stability tests in solution were performed at the level of medium QC samples, with six replicates.

### 2.7. Partial Validation in Mouse Plasma

The selectivity of the analytical methods was determined by the analysis of three different batches of plasma from mice (Janvier Labs, Le Genest-Saint-Isle, France) at the LLOQ defined in the human matrix. Then, the ability of the method to quantify mouse samples with a calibration curve prepared with human plasma was evaluated. Accuracies and precisions of QC samples prepared in mouse plasma were calculated from the human calibration curve. Four independent assays with two QC replicates of the three previous levels were performed. Each calibration curve was validated with QC samples in human plasma prepared independently.

### 2.8. Pharmacokinetic Study in Naive Mice

Nine-week-old male Swiss mice (Janvier Labs, Le Genest-Saint-Isle, France), weighing 35–45 g at the time of experimentation, were used. The animals (three to four per cage) were acclimated for a 14-day period before the beginning of the experiments in an environment maintained at 22 ± 1°C with controlled humidity and a 12-h dark/light cycle (light between 7 a.m. and 7 p.m.). Food and tap water were given ad libitum. All experimental procedures were performed in compliance with the European Directive 2010/63/EU on the protection of animals used for scientific purposes and after approval by the Institutional Animal Care and Research Advisory Committee of the French Army Health Service (authorization *n*°596,880, October 10, 2022).

Twenty-four hours before the experiment, mice were anesthetized using isoflurane gas (Vetflurane, Virbac, France) at a concentration of 1.5%–4.0%. Their hind legs were depilated after a 1 min application of a commercial depilatory cream and rinsed with water. Subsequently, the mice were returned to their cages to allow for recovery and complete anesthesia washout. On the day of the experiment, an initial blood sample (20–30 μL) was collected by perforation of the saphenous vein from the mice. Mice were then intraperitoneally injected (10 mL/kg) with atropine sulfate or tiotropium bromide at the dose of 3 mg/kg (*n* = 10, both).

For each treatment group, mice were subdivided into two cohorts, with blood samples collected at distinct time intervals after injection, totaling 14 time points for pharmacokinetic modeling. Compliance with regulatory directives prevented the collection of more than eight blood samples from individual animals. Accordingly, additional blood samples were obtained from the saphenous vein at 5, 15, 40, 75, 105, 150, and 210 min postinjection for one cohort (*n* = 5), while the other cohort (*n* = 5) had blood samples collected at 2, 10, 25, 60, 90, 120, and 180 min postinjection. Blood samples were acquired using heparinized capillaries containing 2 μL of sodium heparin (Panpharma, France). After collection, the samples were transferred to Eppendorf tubes and centrifuged at 3000 g and 4°C for 10 min. The recovered plasma was then stored at −80°C until analysis. At the end of the experiments, the mice were euthanized by i.p. lethal injection (150 mg/kg) of sodium pentobarbital (Doléthal, Vétoquinol SA, France).

Pharmacokinetic analysis was carried out from the average concentration values at each time point using the Phoenix WinNonLin Version 8.4 software (Certara, Princeton NJ, USA). Pharmacokinetic parameters were determined from the plasma concentration-time data using a compartmental approach and include the total area under the plasma concentration-time curve (AUC_0-∞_), elimination half-life (*t*_1/2elim_), total clearance (CL/*F*), and volume of distribution (*V*). CL and *V* were uncorrected for the bioavailability (*F*).

## 3. Results and Discussion

### 3.1. Selectivity and Matrix Effect

Due to atropine's interference with the tiotropium signal, the validation of the method was conducted separately for both compounds. Since the study did not aim to administer a combination of the two drugs, this was not considered a limitation.

**Figure 2:**
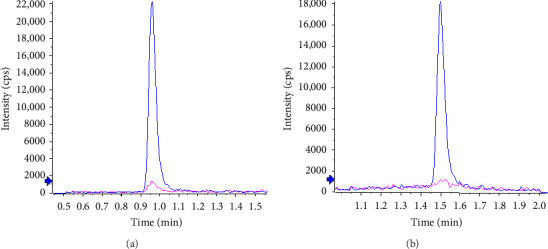
Overlay of typical chromatograms for atropine (a) and tiotropium (b) in human plasma obtained from blank plasma spiked with their internal standard (pink line) and plasma spiked at the LLOQ concentration (blue line).

Typical chromatograms are presented in Figure 2. No substantial interferences were observed at the retention times of the analytes in the plasma samples spiked with IS. Furthermore, no interference at the retention time of IS was observed during validation. The observed retention times were 0.96 min (RSD 0.5%, *n* = 24) for atropine and 1.50 min for tiotropium (RSD 0.4%, *n* = 24). The *k*' values were 3.1 and 5, respectively. Under the specified chromatographic conditions, the number of theoretical plates at the low-level QC was approximately 800 for atropine and 2000 for tiotropium.

No matrix or concentration effects were observed for either analyte. The relative signals were 102.8 ± 6.0% for atropine and 98.1 ± 1.4% for tiotropium.

### 3.2. Drug/Response Relationship

Interday assays were performed over three independent runs conducted on separate days. A linear calibration curve for atropine and a quadratic calibration curve for tiotropium, both weighted by concentration, provided the best fit between nominal and back-calculated concentrations. Determination coefficients were consistently above 0.999.  Table 2 summarizes the mean ± RSD values for back-calculated concentrations, with recoveries ranging from 94.8% to 107.2% and RSD consistently below 9%. Comparison of back-calculated and nominal concentrations using simple linear regression revealed no significant deviation, with slopes and the intercepts not statistically different from 1 and 0, respectively (1.000 IC_95_[0.992–1.007] and 0.019 IC_95_[−0.233–0.270] for atropine and 0.999 IC_95_[0.990–1.008] and −0.005 IC_95_[−0.248–0.239] for tiotropium). A simple linear regression between residuals and nominal concentrations confirmed no correlation, and the residuals exhibited a zero-centered distribution. Slopes and intercepts were not statistically different from 0 (−0.001 IC_95_[−0.006–0.004] and 0.737 IC_95_[−1.304–2.778] for atropine and 0.000 IC_95_[−0.007–0.007] and 0.024 IC_95_[−2.714–2.762] for tiotropium).

### 3.3. Accuracy, Precision, Extraction Recovery, and Carry-Over

Considering within- and between-run variations, precision was below 8% for both compounds, and accuracy ranged from 87% to 110%. Individual results are presented in Table 3. These results are in line with the guidelines. When comparing the signal in samples spiked before and after the extraction, the mean extraction recoveries were 98.3 ± 8.6% and 99.9 ± 8.6% for atropine and tiotropium, respectively. There was also no concentration effect.

No significant carry-over was observed, and the response level immediately following the injection of the ULOQ was always below the recommended limit value (i.e., < 20% of the LLOQ signal). The mean value was 9.4 ± 3.9% for atropine and 12.0 ± 4.4% for tiotropium.

### 3.4. Stability

Analytes in stock and working solutions were stable for at least 4 h at room temperature and 5 days at 4°C. Freeze-thaw analysis results suggested that plasma samples could be thawed and refrozen at least three times without compromising sample integrity.

Benchtop and storage stability tests confirmed that analytes in plasma samples were stable for at least 4 h at room temperature and 24 h at 4°C. The resulting extracted samples were stable for at least 72 h at room temperature and in the auto sampler at 6°C. Long-term stability testing revealed that analytes in plasma samples were stable for at least 3 weeks at −30°C and 2.5 months at −80°C. The results are shown in Table 4.

### 3.5. Partial Validation in Mouse Plasma

**Figure 3:**
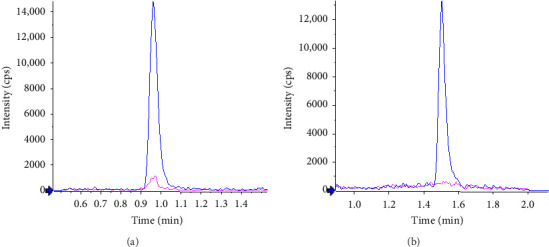
Overlay of typical chromatograms for atropine (a) and tiotropium (b) in mouse plasma obtained from blank plasma spiked with their internal standard (pink line) and plasma spiked at the LLOQ concentration (blue line).

The absence of interference in mouse plasma was observed as well.  Figure 3 shows typical chromatograms obtained from extracts of blank mouse plasma, spiked or not with the analyte at the LLOQ. The selectivity of the method for both compounds was demonstrated by representative chromatograms of blank matrices spiked with the IS. Retention times were not affected by the species change.

Between-run (*n* = 4) accuracy and precision of QC samples prepared in duplicate in different batches of mouse plasma and calculated from calibration curves prepared in human plasma are presented in Table 5. Accuracies ranged from 93.1% to 102.7%, and precision was below 11%. In parallel, calibration curves were validated with QC samples prepared in human plasma. Accuracies were 103.2 ± 7.7% and 102.5 ± 8.2% for atropine and tiotropium, respectively.

Thus, these tests showed no divergence between the two species. It is therefore possible to use human plasma for the determination of the two analytes in mouse plasma.

### 3.6. Pharmacokinetic Studies

Since the murine model did not allow the collection of large plasma volumes, we developed a sensitive method for analyzing microvolumes of plasma. This approach enabled us to model the time-dependent evolution of atropine and tiotropium concentrations in plasma. Abbara et al. [17] notably described a method for quantifying atropine in human plasma with an LLOQ of 0.25 ng/mL using a sample volume of 100 μL. In comparison, our method allows for the quantification of atropine from a sample volume 10 times smaller, with an LLOQ of 1 ng/mL. Regarding tiotropium, Wang et al. [23] developed an HPLC-MS/MS method with a quantification range of 0.5–50 pg/mL for a human plasma sample volume of 400 μL. While this method has greater sensitivity, it requires a sample volume 40 times larger than our method and has a narrow quantification range, making it unsuitable for our samples, which are more concentrated or require prior dilution. Our method, with a quantification range of 0.5–1–1000 ng/mL, is therefore well-suited to our experimental constraints.

**Figure 4:**
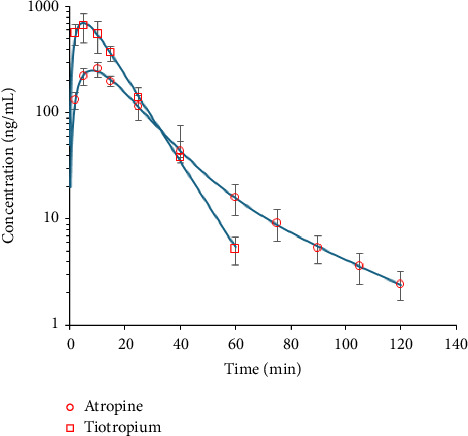
Mean plasma concentration versus time curves after intraperitoneal administration of 3 mg/kg of atropine sulfate (circle) or tiotropium bromide (square) in the naive mouse. The observed data are expressed in red, and the predicted data are expressed in blue.

Semilogarithmic plots of the mean (±SD) plasma concentration-time profiles are illustrated in Figure 4. Concentrations were below the LLOQ after 60 and 120 min for tiotropium and atropine, respectively. The data were fitted to a one-compartment model for tiotropium and a two-compartment model for atropine. Maximum concentrations were reached 5–10 min after drug administration: 8.18 ± 0.22 min for atropine and 4.89 ± 0.24 min for tiotropium. Tiotropium exhibited a higher maximum concentration compared to atropine (705 ± 22 ng/mL vs. 251 ± 5 ng/mL) and a higher AUC (11,920 ± 289 min.ng/mL vs. 6764 ± 98 min.ng/mL). Atropine showed a larger volume of distribution (5204 ± 430 mL/kg vs. 2158 ± 75 mL/kg), and its elimination half-life was slightly longer than tiotropium's (9.76 ± 0.77 min vs. 7.43 ± 0.12 min). A summary of these results is shown in Table 6.

To our knowledge, pharmacokinetic modeling of these compounds has not been previously established in mice. For atropine, Kentrop et al. [24] described a one-compartment pharmacokinetic profile in a guinea pig model following an intramuscular injection (0.4 mg/kg), which closely aligns with our findings; particularly, the *T*_max_ reached in less than 10 min.

The pharmacokinetics of these compounds highlight a key advantage of antimuscarinics: their rapid diffusion into the bloodstream. This property justifies their use as emergency treatments for OP poisoning, where atropine is administered at the first signs of toxicity. However, due to their rapid elimination, successive doses must be administered to maintain effective concentrations. Therefore, pharmacokinetic modeling of these compounds in our mouse model will allow us to optimize the therapeutic regimen for the administration of atropine and tiotropium.

## 4. Conclusion

In this study, a quantitative LC-MS/MS method was developed for the determination of atropine and tiotropium. The literature does not report any method for quantifying these compounds in very small sample volumes (at least 100–300 μL). Therefore, we designed and validated a method, in accordance with ICH M10 guidelines, that is suitable for our mouse model, which does not allow for successive collection of large plasma volumes.

Given the experimental constraints of working with a maximum of 10 μL of plasma per sampling time in mice, the method was optimized to ensure high sensitivity within these limited volumes. Using only 10 μL of plasma, the sensitivity obtained was in the order of ng/mL. The LLOQ were 1 and 0.5 ng/mL for atropine and tiotropium, respectively. This level of sensitivity enabled the withdrawal of five time-point samples per subject during the pharmacokinetic study. Thus, the method has contributed to reducing the number of mice used.

With this consideration, the method was first validated in human samples according to the recommended guidelines. For both compounds, the goodness of fit between back-calculated and nominal concentrations was demonstrated, no matrix effect was observed, and a high level of extraction recovery was achieved. Then, a partial validation was performed, showing good precision and accuracy with a QC sample in a murine matrix quantified with calibration curves in human plasma.

The SPE method described in this paper can be easily automated, allowing a fast turnaround time. This results in a high throughput capability, which is favorable for pharmacokinetic/pharmacodynamic applications. This method was applied to the determination of pharmacokinetic parameters of atropine and tiotropium in naive mice. This will subsequently enable the proposal of therapeutic schemes for an OP-intoxicated model.

## Figures and Tables

**Table 1:** Detection parameters of atropine, tiotropium, and their internal standard.

Compound	Precursor (*m*/*z*)	DP (*V*)	EP (*V*)	Quantifier			Qualifier		
				Product ion (*m*/*z*)	CE (*V*)	CXP (*V*)	Product ion (*m*/*z*)	CE (*V*)	CXP (*V*)
Atropine	290.0	160	10	124.0	32	14	93.0	37	14
Atropine-d_5_	295.2			124.1					
Tiotropium	392.0			152.1	35	10	170.2	40	15
Tiotropium-d_3_	395.0			155.0			173.0		

*Note:* CXP, collision cell exit potential; DP, declustering potential.

Abbreviations: CE, collision energy; EP, exit potential.

**Table 2:** Recovery from back-calculated concentrations and relative standard deviation (R.S.D) from calibration curves for atropine and tiotropium in human plasma (*n* = 3).

Theoretical concentration (ng/mL)	Recovery (%)		R.S.D. (%)	
	Atropine	Tiotropium	Atropine	Tiotropium
0.50	/	107.2	/	8.3
1.00	105.4	/	4.8	/
2.50	98.1	100.1	6.1	2.3
5.00	98.5	97.6	0.9	2.4
25.0	100.5	94.8	6.1	8.5
100	100.3	99.8	1.7	0.7
250	102.5	99.6	1.9	3.7
500	98.6	101.2	1.7	2.3
1000	100.0	99.5	0.6	0.6

**Table 3:** Within (*n* = 5) and between (*n* = 12) run accuracy and precision of the method in human plasma.

Theoretical concentration (ng/mL)	Accuracy (%)		Precision (%)	
	Atropine	Tiotropium	Atropine	Tiotropium
*Within-run*				
0.50	/	107.1	/	2.2
1.00	87.4	/	4.4	/
1.50	96.3	98.9	6.3	6.3
50.0	93.7	97.1	5.9	2.8
750	96.2	103.8	4.9	4.5

*Between-run*				
0.50	/	109.5	/	6.3
1.00	96.9	/	6.3	/
1.50	96.2	103.4	7.4	4.9
50.0	95.9	97.4	5.5	3.3
750	96.4	101.6	5.1	6.8

**Table 4:** Freeze and thaw stability, bench-top stability, and processed sample stability of atropine and tiotropium in human plasma.

Stability evaluation (*n* = 3)	Conditions		Accuracy (%) ± precision (%)			
	Temperature	Time	Theoretical concentrations (ng/mL)			
			1.5		750	
			Atropine	Tiotropium	Atropine	Tiotropium
Freeze and thaw stability	−80°C	3 cycles of 24 h	100.4 ± 12.3	114.7 ± 1.4	106.2 ± 3.7	99.9 ± 1.7

Bench-top stability in plasma	Room temperature	4 h	96.6 ± 4.8	101.4 ± 1.8	99.1 ± 2.7	96.7 ± 2.9

Conservation stability in plasma	4°C	24 h	85.0 ± 6.6	114.0 ± 6.6	99.4 ± 4.8	92.4 ± 4.1
	−30°C	3 weeks	89.9 ± 7.4	110.1 ± 5.6	99.2 ± 1.4	112.5 ± 2.0
	−80°C	3 months	107.9 ± 4.3	99.6 ± 1.8	111.4 ± 5.3	105.2 ± 1.2

Extract stability	Room temperature	72 h	97.6 ± 3.7	111.5 ± 1.9	97.3 ± 1.8	99.0 ± 3.4
	6°C		94.5 ± 1.3	88.2 ± 4.9	100.8 ± 2.1	98.4 ± 0.8

**Table 5:** Between-run accuracy and precision on QC samples in mouse plasma calculated with calibration curves prepared in human plasma (*n* = 8).

Theoretical concentration (ng/mL)	Accuracy (%)		Precision (%)	
	Atropine	Tiotropium	Atropine	Tiotropium
1.50	93.1	102.7	6.9	10.9
50.0	93.3	93.4	5.7	6.2
750	98.6	97.9	8.6	7.9

**Table 6:** Pharmacokinetic parameters for intraperitoneally administrated atropine sulfate and tiotropium bromide in naïve mice.

Parameter	Units	Atropine	Tiotropium
AUC	min.ng/mL	6764 ± 98	11,920 ± 289
*T*_max_	min	8.18 ± 0.22	4.89 ± 0.24
*C*_max_	ng/mL	251 ± 5	705 ± 22
*t*_1/2elim_	min	9.76 ± 0.77	7.43 ± 0.12
CL_F	mL/min/kg	370 ± 5.4	201 ± 5
V_F	mL/kg	5204 ± 430	2158 ± 75

*Note:* For each parameter, the mean value is associated with the standard deviation of the model.

## Data Availability

The data that support the findings of this study are available from the corresponding author upon reasonable request.
